# The *Leishmania infantum *PUF proteins are targets of the humoral response during visceral leishmaniasis

**DOI:** 10.1186/1756-0500-3-13

**Published:** 2010-01-21

**Authors:** Cristina Folgueira, Marta Martínez-Bonet, Jose M Requena

**Affiliations:** 1 Centro de Biología Molecular Severo Ochoa (CSIC-UAM), Universidad Autónoma de Madrid, Madrid, Spain

## Abstract

**Background:**

RNA-binding proteins of the PUF family share a conserved domain consisting of tandemly repeated 36-40 amino acid motifs (typically eight) known as Puf repeats. Proteins containing tandem repeats are often dominant targets of humoral responses during infectious diseases. Thus, we considered of interest to analyze whether *Leishmania *PUF proteins result antigenic during visceral leishmaniasis (VL).

**Findings:**

Here, employing whole-genome databases, we report the composition, and structural features, of the PUF family in *Leishmania infantum*. Additionally, the 10 genes of the *L. infantum *PUF family were cloned and used to express the *Leishmania *PUFs in bacteria as recombinant proteins. Finally, the antigenicity of these PUF proteins was evaluated by determining levels of specific antibodies in sera from experimentally infected hamsters. The *Leishmania *PUFs were all recognized by the sera, even though with different degree of reactivity and/or frequency of recognition. The reactivity of hamster sera against recombinant LiPUF1 and LiPUF2 was particularly prominent, and these proteins were subsequently assayed against sera from human patients. High antibody responses against rLiPUF1 and rLiPUF2 were found in sera from VL patients, but these proteins resulted also recognized by sera from Chagas' disease patients.

**Conclusion:**

Our results suggest that *Leishmania *PUFs are targets of the humoral response during *L. infantum *infection and may represent candidates for serodiagnosis and/or vaccine reagents; however, it should be kept in mind the cross-reactivity of LiPUFs with antibodies induced against other trypanosomatids such as *Trypanosoma cruzi*.

## Findings

Leishmaniases constitute a vast variety of diseases ranging from cutaneous manifestations to visceral dissemination that are caused by different species of the genus *Leishmania*. Visceral leishmaniasis (VL), the most severe form of leishmaniases (usually fatal if untreated), is typically caused by *Leishmania donovani *and *Leishmania infantum *(syn. *Leishmania chagasi*) [1]. Leishmaniases constitute a worldwide public-health problem and 1.5 to 2 million new cases occur every year: 1 to 1.5 million cases of cutaneous forms and 500,000 cases of VL [2]. These vector-borne diseases are endemic in large areas of the tropics, subtropics and the Mediterranean basin. Early and accurate diagnosis of leishmaniasis remains unresolved. Diagnostic tests should be highly sensitive and specific; furthermore, the tests should be able to make the distinction between acute disease and asymptomatic infection. Also, accurate diagnosis is relevant for monitoring success in the chemotherapeutic treatment of leishmaniases.

Parasite detection in tissue biopsies either by microscopic visualization of amastigotes or in vitro culturing of the parasite remains the gold standard in leishmaniasis diagnosis because of its high specificity [1,3]. However, for VL diagnosis, aspirates of lymph nodes, bone marrow or spleen need to be obtained, being both risky and painful for patients. In addition, the sensitivity of microscopy and culture techniques tends to be low and variable, depending of many factors, among them the technical skills of the personnel. Given that leishmanial infections are accompanied by a dramatic humoral response, mainly in the visceral forms of leishmaniasis, serodiagnosis appears as an interesting alternative. In fact, a battery of immunological procedures have been developed and adapted for diagnosis and epidemiological surveys [4]. Several *Leishmania *antigenic proteins have been expressed as recombinant proteins and their potential for serodiagnosis assayed [5-8]. However, so far, only a *Leishmania *recombinant protein (rK39) is being used for serodiagnosis of leishmaniasis in the field [1]. rK39 is a subfragment within a 230-kDa kinesin-related protein (LcKin); it spans the LcKin C-terminal region, which contains seven 39-amino acid repeats [9]. Many antigens of different protozoan parasites contains tandem repeats in their structure [10,11], suggesting that repeated domains might be better inducers of B-cell activation and, therefore, resulting preferred targets of the humoral responses. Experimental support for this hypothesis has been obtained by Goto and co-workers [12]. By screening an *L. infantum *expression library with sera from human VL patients or infected hamsters, they identified 43 genes encoding B-cell antigens, and, remarkably, 44% of those antigens contained tandem repeats in their structure. In a subsequent work, these authors, using a bioinformatic approach, directly searched in the *L. infantum *database for genes encoding proteins with tandem repeats. Among the 64 identified genes, the antigenicity for 6 of the encoded proteins was experimentally assayed using sera from VL patients, showing that 4 of them had significant and specific reactivity against VL sera [13].

The RNA-binding proteins of the PUF family constitute an evolutionarily conserved group of proteins present in all eukaryotic phyla [14]. PUF proteins bind to sequence motifs in the 3'-UTR of specific mRNAs and control their localization, stability and translation [15]. The distinctive feature of PUF proteins is the RNA-binding domain known as the Pumilio homology domain (PUM-HD), which is composed of several consecutive imperfect repeats (typically eight) of around 36-40 amino acids (Puf repeats). Recently, Caro and co-workers [16] identified up to 10 different PUF proteins in the genome of *Trypanosoma cruzi*, and an equivalent complement of PUF proteins has been also described for two other trypanosomatids, *Trypanosoma brucei *and *Leishmania major *[17]. Because of the existence of many repeated motifs in this class of proteins, we questioned whether the PUF proteins would be antigenic during *Leishmania *infection. For this purpose, we have identified the putative *PUF *genes existing in the *L. infantum *genome and expressed them as recombinant proteins. Afterwards, the antigenicity of the different *L. infantum *PUFs was assayed using the sera from experimentally infected hamsters. Interestingly, all the PUFs were specifically recognized by immune sera, even though individual differences were observed.

## Methods

### Parasites and sera

*L. infantum *promastigotes were cultured at 26°C in RPMI containing 10% heat-inactivated, fetal bovine serum. Hamsters were infected intracardially with stationary-phase promastigotes (strain M/CAN/ES/96/BNC150) and the animals were monitored for 1-year follow-up period. At the onset of clear symptoms of visceral leishmaniasis (emaciation and asthenia) hamsters 1, 2, 3, 4, 5, 7 and 8 were sacrificed; high parasite loads were found in both liver and spleen of these animals. At the end of the study, even though hamsters 9 and 10 did not show external signs of disease, significant parasite loads were found in their livers and spleens. Hamster 6 was a particular case, no parasites were observed after microscopic examination of liver or spleen preparations at the moment of sacrifice; thus, it was assumed that this animal had resolved the infection. Before infection (preimmune serum) and at the time of sacrifice (immune serum), sera were collected. See reference [18] for further details of this infection experiment (code correspondence: 1 = A2, 2 = A3, 3 = A4, 4 = B1, 5 = B3, 6 = B4, 7 = C3, 8 = C4, 9 = A5, 10 = A7). All experimental animals were housed, fed and used in accordance with the Federation of European Laboratory Animal Science Association (FELASA) Guidelines. The animal study protocol was approved by the Ethics Committee of the Universidad Autónoma de Madrid.

Human sera were provided by Dr. Carmen Cañavate (WHO Collaborating Centre for Leishmaniasis, Servicio de Parasitología, Centro Nacional de Microbiología, Madrid). VL sera were collected from Spanish patients with active disease that was diagnosed by serological and parasitological methods. Chagasic sera were obtained from Latin American immigrants showing signs of cardiomyopathy and proven serologically positive by IFI and commercial ELISA kits. Normal sera were obtained from healthy individuals in Spain.

### Identification and bioinformatic analyses of the *L. infantum *PUF proteins

*T. brucei*, *T. cruzi*, *L. braziliensis*, *L. infantum *and *L. major *PUF sequences were obtained from GeneDB [19]. Searches for structural motifs in *Leishmania *PUF sequences were performed using the PROSITE database and resources [20]. Potential N-terminal signal peptides were predicted by Signal P program [21].

### Cloning of *L. infantum *PUF genes and expression of recombinant proteins

The complete coding sequences of LinJ06_V3.1830, LinJ11_V3.0470, LinJ12_V3.0330, LinJ18_V3.1400, LinJ20_V3.1420, LinJ21_V3.2050, LinJ25_V3.2470, LinJ32_V3.1830 and LinJ33_V3.1210 genes were amplified by PCR. The sequences of the oligonucleotide primers used in this study are listed in the Additional file 1. As template, genomic DNA from *L. infantum *JPC strain (MCAN/ES/98/LLM-724) was used.

The PCR products were digested with restriction enzymes (specific restriction sites were added to the 5'-end of the primers) and cloned directionally in the appropriate expression vector (pQE30, pQE31 or pQE32; Qiagen). The sequences of the inserts were determined by automatic sequencing (Servicio de Genómica, Parque Tecnológico de Madrid-UAM). The expression clones were used to transform *Escherichia coli *M15-pREP4 strain bacteria. The optimum conditions for expression were determined by varying the IPTG (isopropyl-β-D-thiogalactopyranose) concentration, length of induction and growth temperature. The recombinant proteins were then purified by Ni-nitrilotriacetic acid agarose chromatography, using denaturing conditions according to the manufacturer's protocol (Qiagen). The purity of the proteins was assayed by sodium dodecyl sulfate-polyacrylamide gel electrophoresis and Coomassie blue staining of the gels. The concentration of proteins was determined by the Bradford dye assay (Bio-Rad) using bovine serum albumin (BSA) as standard.

### Enzyme-linked immunosorbent assay (ELISA)

The recombinant PUF proteins were analyzed for seroreactivity using sera from 10 hamsters with VL symptoms that were experimentally infected with *L. infantum *promastigotes (see above). Preimmune sera from the same animals were used as negative controls. In addition, *L. infantum *soluble lysate antigen (SLA) was used as a positive control. Each sample was assayed in triplicate. Proteins were diluted in coating buffer (15 mM Na_2_CO_3_, 28 mM NaHCO_3_, pH = 9.6), and plates were coated overnight at 4°C with 50 ng of SLA or 200 ng of recombinant protein. After removing the coating solution, free binding sites were blocked with 200 μl of 5% milk in PBS containing 0.3% Tween 20 (T-PBS) for 1 h at room temperature. The plates were then washed two times in T-PBS. Sera diluted 1:200 in T-PBS and 0.5% nonfat milk were added (100 μl/well), and plates were incubated at room temperature for 1 h. After three washes, horseradish peroxidase-conjugated rabbit anti-hamster IgG (Nordic Immunological Lab.), diluted 1:1500 in T-PBS and 0.5% nonfat milk were added (100 μl/well). After incubation at room temperature for 1 h, plates were washed three times. Finally, the antibody binding was revealed with ortho-phenylenediamine (OPD) and hydrogen peroxide. The reaction was left to proceed for 15 min and then stopped by addition of 100 μl of H_2_SO_4 _1 M. The absorbance of the reaction was measured at 450 nm in an ELISA reader (Bio-Rad). The lower limit of positivity (cutoff) was determined by the mean of the negative controls (pre-immune sera) plus three standard deviations. Human sera were also assayed in ELISA at 1:200 dilution, using horseradish peroxidase-conjugated goat anti-human IgG (Nordic Immunological Lab.) as secondary antibody (1:1500 dilution).

## Results and Discussion

### *L. infantum *has an equivalent complement of PUF proteins to those predicted in other trypanosomes

In previous studies, it was uncovered the existence of 10 different PUF proteins (PUF1 to PUF10) in the genomes of *T. cruzi *[16] and *T. brucei *[17]. Homologue proteins were also identified in the *L. major *genome [17], indicating a high degree of conservation of the PUF family in trypanosomatids. Recently, the genome sequences for two additional *Leishmania *species (i.e., *L. infantum *and *L. braziliensis*) have been completed [22]. With this wealth of information, we undertook a comparative analysis of this protein family looking for conserved and divergent features among the different trypanosomatids. As depicted in Table 1, all species have an equivalent complement of PUF proteins, with the intriguing exception of PUF9. This protein is not predicted in the current *L. braziliensis *genome database. However, a BLAST search in the unassembled shotgun reads of *L. braziliensis *Genome project [19] identified sequences with significant homology to *PUF9 *genes (data not shown), suggesting that this gene does exist in *L. braziliensis *but that it has not been annotated yet due to problems in genome coverage.

**Table 1:** Putative PUF proteins in pathogenic trypanosomatids

	*T. cruzi*	*T. brucei*	*L. braziliensis*	*L. infantum*	*L. major*
**Name**	ID^a^	ID	ID	ID	ID

**PUF1**	Tc00.1047053508625.160	Tb10.70.2800	LbrM35_V2.0090	LinJ36_V3.0050	LmjF36.0050

**PUF2**	Tc00.1047053507831.110	Tb10.389.0940	LbrM18_V2.1450	LinJ18_V3.1400	LmjF18.1420

**PUF3**	AY373520^b^	Tb10.100.0190	LbrM21_V2.1990	LinJ21_V3.2050	LmjF21.1680

**PUF4**	Tc00.1047053510073.30	Tb927.6.820	LbrM12_V2.0400	LinJ12_V3.0330	LmjF12.0380

**PUF5**	Tc00.1047053507521.110	Tb927.7.4730	LbrM06_V2.0050	LinJ06_V3.0050	LmjF06.0050

**PUF6**	Tc00.1047053510125.10	Tb10.26.0140	LbrM33_V2.1360	LinJ33_V3.1210	LmjF33.1150

**PUF7**	Tc00.1047053511715.100	Tb11.01.6600	LbrM32_V2.1920	LinJ32_V3.1830	LmjF32.1750

**PUF8**	Tc00.1047053508479.120	Tb927.3.2470	LbrM25_V2.1950	LinJ25_V3.2470	LmjF25.2360

**PUF9**	Tc00.1047053506563.10	Tb927.1.2600	N.A.^c^	LinJ20_V3.1410^d^ LinJ20_V3.1420^e^	LmjF20.1365^d^ LmjF20.1370^e^

**PUF10**	Tc00.1047053506773.130	Tb11.02.4570	LbrM11_V2.0150	LinJ11_V3.0470	LmjF11.0470

^a^GeneDB accession number

^b^GenBank accession number

^c^Not annotated in the *L. braziliensis *genome database

^d^PUF9a

^e^PUF9b

On the other hand, it is remarkable the existence of two isoforms for PUF9 in *L. major *and *L. infantum*. We named these isoforms as PUF9a (LmjF20.1365 and LinJ20_V3.1410; see Table 1) and PUF9b (LmjF20.1370 and LinJ20_V3.1420). Phylogenetic analysis (not shown) suggests that PUF9a/b isoforms are derived from a recent duplication of the ancestral PUF9 gene that occurred in the *L. infantum*/*L. major *evolutionary line. In fact, both genes are contiguous in the *L. infantum*/*L. major *chromosome 20. However, multiple alignment of the sequences for the *Leishmania *PUF9 isoforms evidenced a conspicuous structural difference between *L. infantum *and *L. major *PUF9 proteins: *L. infantum *LiPUF9 isoforms have an N-terminal extension that is absent from the *L. major *counterparts. However, since the sequences upstream of the assigned start codons for LmjPUF9a and LmjPUF9b are extremely conserved with the sequences coding for the N-terminal regions of the LiPUF9a and LiPUF9b (data not shown), the differences in the predicted sequences may be due to annotation errors in the *L. major *database. In fact, even though with low sequence conservation, the *T. brucei *and *T. cruzi *PUF9 proteins have N-terminal extensions equivalent in size to those existing in the *L. infantum *isoforms.

### Structural features of the *L. infantum *PUF proteins

**Figure 1:**
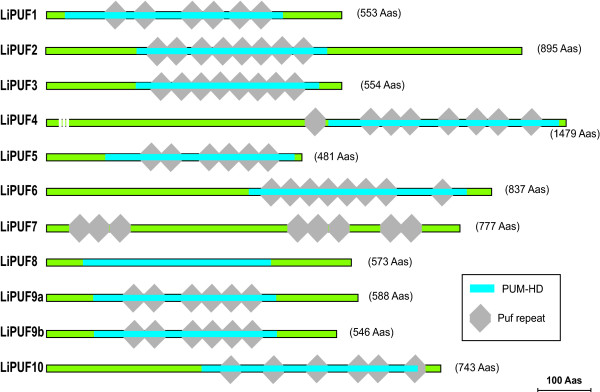
**Diagrammatic representation of structural motifs found in the *L. infantum *PUF proteins**. The positions of the Puf repeats and the PUM-HD domain are indicated.

A common feature of PUF proteins is the presence of an RNA-binding domain, known as the Pumilio homology domain (PUM-HD), which is composed of several consecutive imperfect repeats (Puf repeats; typically eight), each of approximately 40 amino acids [15]. A search for functional domains and structural motifs was performed in the different PUF proteins by scanning the sequences against PROSITE patterns [20]. As shown in figure 1, the PUM-HD domain [Swiss-Prot: PS50303] was predicted for 10 out of the 11 *L. infantum *PUF proteins. The sole exception was LiPUF7 protein, for which the program failed to predict a PUM-HD domain. However, in the *T. brucei *orthologue, TbPUF7, this domain is readily predicted at the N-terminal end of the sequence (data not shown). Additionally, the LiPUF7 possesses a cluster of five PUF repeats at the C-terminal end, suggesting that two RNA-binding domains might be present in this protein (figure 1). In summary, from these bioinformatics analyses, it became evident that PUF proteins of *Leishmania *are rich in repeated motifs, i.e, Puf repeats.

### Recognition of the *L. infantum *PUF proteins by sera from experimentally infected hamsters

The repeated nature of PUF proteins prompted us to analyze whether, as hypothesized by other authors [13], they can be targeted by the immune system during *Leishmania *infection. In order to determine the antigenicity of the *L. infantum *PUF proteins, we cloned the complete coding regions of 10 out of the 11 PUF genes existing in this *Leishmania *species (Table 1). Since LiPUF9a and LiPUF9b are essentially identical in sequence and, presumably, antigenically cross-reactive, we selected one gene (i.e. *LiPUF9b*) for cloning and expression. The different PUF genes were successfully expressed as His-tagged recombinant proteins in *E. coli *(see Methods for details). After affinity chromatography purification, the recombinant PUF (rPUF) proteins were assayed by SDS-polyacrylamide gel electrophoresis, showing apparent molecular masses similar to those deduced from the amino acid sequence [see Additional file 1].

**Figure 2:**
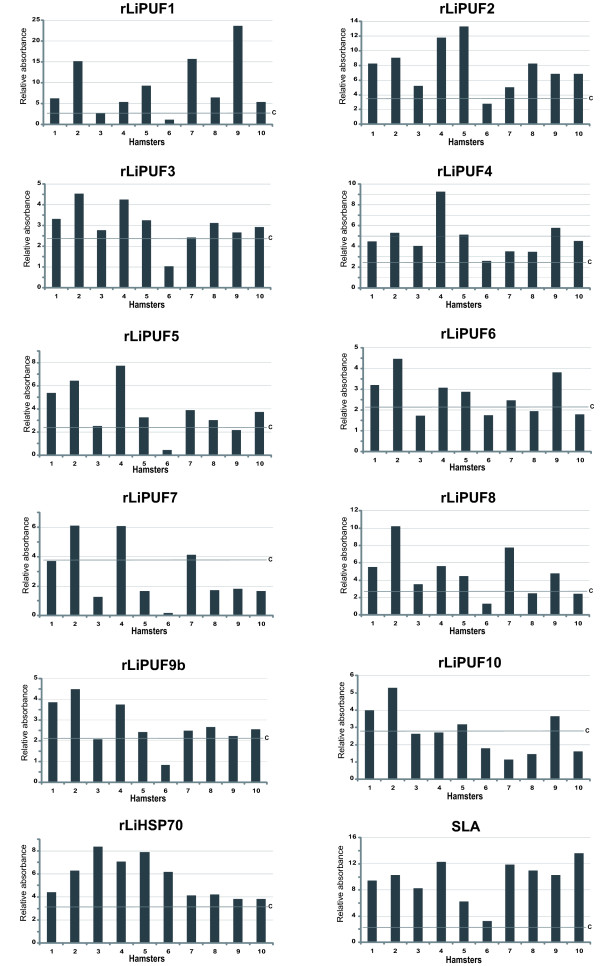
**Recognition of the *L. infantum *PUF proteins by sera from experimentally infected hamsters**. Sera (n = 10) were individually tested for reactivity to rLiPUF1-10, rHSP70 and SLA. C-labeled lines represent the cut-off value, calculated as the mean plus 3 SD of the values for pre-infection sera. Relative absorbance = absorbance value of test serum ÷ mean of the absorbance of sera from animals before infection (pre-inmune sera).

**Figure 3:**
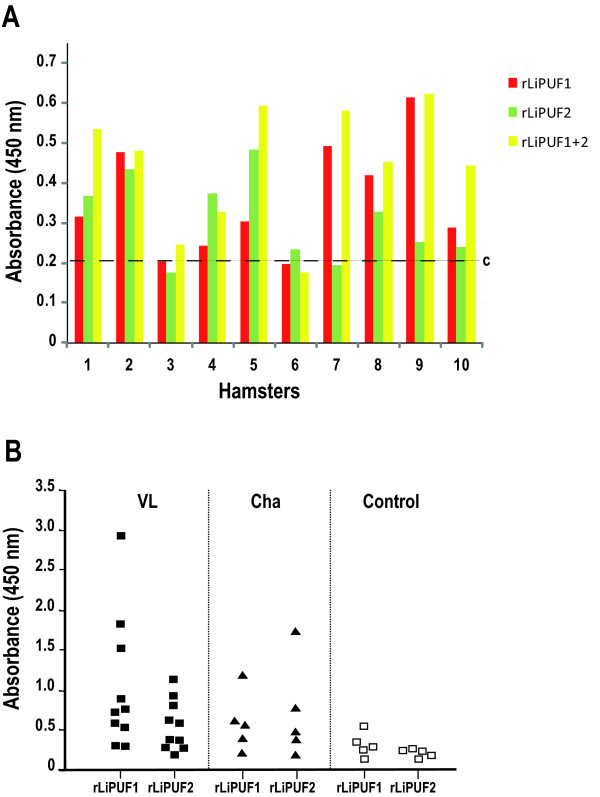
**Analysis of IgG reactivity against rLiPUF1 and rLiPUF2**. (A) The reactivity of sera from the experimentally infected hamsters was determined by ELISA against rLiPUF1, rLiPUF2 and a mixture of both. (B) ELISA reactivity of sera from patients with VL (n = 10) or Chagas' disease (Cha, n = 5) and healthy controls against rLiPUF1 and rLiPUF2. Cut-off value = 0.49.

We then examined the presence of antibodies against the different rPUFs in sera from 10 hamsters with visceral leishmaniasis [18]. The reactivity of pre-infection sera was also analyzed against each one of the recombinant proteins and used to determine the corresponding cut-off values (figure 2). For comparison, we analyzed the reactivity of hamster sera against *L. infantum *soluble proteins (SLA) and rLiHSP70 (a prominent antigen during VL [23]). The individual reactivity of the immune sera against each one of the recombinant PUFs is depicted in figure 2. Remarkably, even though with different degree of reactivity, all ten rLiPUFs were specifically recognized by the different VL sera, suggesting that they are immunogenic for the hamster immune system. Two, rLiPUF1 and rLiPUF2, showed strong reactivity to the VL sera with higher reactivity values than that of SLA. rLiPUF4, rLiPUF5 and rLiPUF8 showed intermediate reactivity, with values similar to that of rLiHSP70. The other PUF proteins (rLiPUF3, rLiPUF6, rLiPUF7, rLiPUF9b and rLiPUF10) showed a lower recognition, but they were specifically recognized by some of the VL sera. An idea arose from these results was the possible combination of several LiPUFs to increase the serodiagnosis usefulness of these proteins. Thus, coating of ELISA plates with a mixture of rLiPUF1 and rLiPUF2 resulted in an overall increase of the reactivity values of sera relative to the values obtained against the proteins assayed separately (figure 3A).

Another remarkable finding of this study is that the PUF antigens are differentially recognized by each one of the hamster sera. Thus, for example, rLiPUF1 was highly recognized by serum from animals 2, 7 and 9, whereas rLiPUF2 was recognized better by sera 4 and 5. Additional variations can be easily deduced from a visual inspection of figure 2. Individual differences in the antigens targeted by the immune system are not unexpected findings, at least, for non inbred animals [24]. Furthermore, this variability of recognition indicates that different LiPUFs are priming specific humoral responses in hamsters, and that the cross-reactivity of the elicited anti-PUF antibodies seems to be low.

### Antigenicity and specificity of rLiPUF1 and rLiPUF2 in human patients

Given the prominent recognition of rLiPUF1 and rLiPUF2 by sera from *L. infantum*-infected hamsters, we assayed the reactivity of 10 sera from VL patients against each one of these recombinant proteins (figure 3B). Remarkably, 8/10 and 5/10 of the VL sera reacted with rLiPUF1 and rLiPUF2, respectively, with absorbance values higher than the cut-off value of the healthy controls (0.49). Some of the human VL sera yielded absorbance values higher than 1, indicating that these proteins are strong immunogens for the immune system of some individuals. Since the LiPUF proteins have well-conserved homologues in other trypanosomatids (Table 1), we further analyzed whether specific antibodies are present in the sera from Chagas' disease patients. Not surprisingly, positive reactivity against rLiPUF1 and rLiPUF2 was observed in 3/5 and 2/5, respectively, of the sera from chagasic patients. This cross-reactivity may be a hurdle for the use of these two proteins as diagnostic antigens, at least in regions where leishmaniasis and Chagas's disease are endemic. Nevertheless, they should not be absolutely discarded before determining the location of B-cell epitopes recognized by antibodies elicited in VL and chagasic patients. For example, acidic ribosomal proteins are prominent and cross-reactive antigens during both leishmaniasis and Chagas' disease, but the recognized epitopes are different for each group of patients, allowing that a truncated form of the *Leishmania *P2 protein could be used for differential serodiagnosis of these diseases [25].

In order to evaluate the diagnosis usefulness of these proteins, it remains to determine the recognition of the *Leishmania *PUFs by sera from patients with different forms of leishmaniasis and/or VL dogs. Currently, we are planning those studies with the hope that some of these proteins can be used together with other well characterized *Leishmania *antigens for designing new serodiagnosis devices. Diagnostic arrays based on antigens that most often are targeted by human immune system are expected to be far more sensitive and informative than simple monospecific diagnostics such as the currently widely applied rK39 dipstick, as single antigens that are recognized by all individuals infected by any pathogen appear to be rare.

## Conclusions

*L. infantum *shares with other *Leishmania *species and related trypanosomatids (e.g. *T. brucei *and *T. cruzi*) a PUF family consisting of ten members. All ten *L.infantum *PUF are B-cell antigens during parasite infection in hamster model of VL, even though with variable strength. Thus, rLiPUF1 and rLiPUF2 showed strong reactivity, rLiPUF4, rLiPUF5 and rLiPUF8 showed intermediate reactivity, whereas rLiPUF3, rLiPUF6, rLiPUF7, rLiPUF9b and rLiPUF10 showed low reactivity. We also showed that rLiPUF1 and rLiPUF2 are recognized by sera from VL patients, but also by sera from Chagas' disease patients. In conclusion, this study has shown that PUFs are targets of the immune system during *L. infantum *infection and, therefore, might have usefulness in diagnosis and/or vaccine developments.

## Competing interests

The authors declare that they have no competing interests.

## Authors' contributions

CF and JMR conceived and designed the experiments. CF performed the cloning and expression experiments. MMB carried out the ELISA experiments. JMR analyzed the data and wrote the paper. All authors read and approved the final manuscript.

## Supplementary Material

Additional file 1 **Oligonucleotides used for cloning of PUF genes**. This file contains the nucleotide sequences of the primers used for PCR amplification of the *L. infantum PUF *genes. Molecular masses of the recombinant proteins, as deduced from the nucleotide sequence in the expression vectors, are also found in this file. Click here for file
