# Supplementary material for: The Leishmania infantum PUF proteins are targets of the humoral response during visceral leishmaniasis
Source: BMC Res Notes. 2010 Jan 21;3:13. doi: 10.1186/1756-0500-3-13 (PMC2830943; doi:10.1186/1756-0500-3-13)
Supplement: Additional file 1 — Oligonucleotides used for cloning of PUF genes. This file contains the nucleotide sequences of the primers used for PCR amplification of the L. infantum PUF genes. Molecular masses of the recombinant proteins, as deduced from the nucleotide sequence in the expression vectors, are also found in this file. [file 1756-0500-3-13-S1.PDF]

Table S1. Oligonucleotides used for cloning of PUF genes and molecular masses of the recombinant proteins.

Name	Molecular mass[a]	Primers used for PCR-amplification of the gene[b]	GeneDB identifier[c]
**rLiPUF1**	64085,15 Da	F: GAATTCGTCAGAGGAGAAGTTGAC<br>R: CTCGAGTTAGCGGTACTCAGGCAGGC	LinJ36_V3.0050
**rLiPUF2**	98172,83 Da	F: GGATCCATGTCTTCTAACTGGACTGCC<br>R: AAGCTTCTAGTAGCCCGTCTCCTGCT	LinJ18_V3.1400
**rLiPUF3**	67335.14 Da	F: GGCCCGGGATGGCCTGGACGGTGCACGAG<br>R: CCTCTAGAACGCCGTCCGTGGTTGGGGGA	LinJ21_V3.2050
**rLiPUF4**	156451,80 Da	F: GGATCCATGTTCGCCCAATCCGAGAG<br>R: AAGCTTCAACTGCGGCGCTGCTTCT	LinJ12_V3.0330
**rLiPUF5**	54042,24 Da	F: GGATCCATGTCTGCCGGTTCGAAGAA<br>R: GTCGACTACTTGTTCATGGCATCCACT	LinJ06_V3.0050
**rLiPUF6**	93224,62 Da	F: GGATCCTGTACTCGGAACAGAGCTGG<br>R: GATATCTTAGCGGCGGTGGTGGTTGG	LinJ33_V3.1210
**rLiPUF7**	86017,57 Da	F: GGATCCATGCCGGGAATGAAGCTGGA<br>R: AAGCTTCTACTTCTTCATAGAGCGGAAGAG	LinJ32_V3.1830
**rLiPUF8**	66095,51 Da	F: GGATCCATGGGCCGTACGAATGCGAA<br>R: GTCGACTAAATCTTCTTGACCCTCTCC	LinJ25_V3.2470
**rLiPUF9b**	59796,86 Da	F: GGATCCATGTCTTCGTCTGCCGAGCG<br>R: GTCGACTACTTTGAGAGCGGCTGCCG	LinJ20_V3.1420
**rLiPUF10**	82155,28 Da	F: GGATCCATGGGCAAGAAGGCGGAGTT<br>R: AAGCTTCTACTGGGGTTGCTCCTGTTCCG	LinJ11_V3.0470

[a]Molecular mass of the recombinant protein deduced from the nucleotide sequence in the expression vector
[b]F, forward; R, reverse
[c] GeneDB [19]
